# Perioperative Management of Patients using GLP-1 Receptor Agonists Current Evidence, Risks, and Practical Recommendations-A Narrative Review

**DOI:** 10.4274/TJAR.2026.262459

**Published:** 2026-06-26

**Authors:** Uğur Serkan Çitilcioğlu, Hatice Kaya Özdoğan

**Affiliations:** 1 University of Health Sciences Türkiye, Adana City Hospital, Clinic of Anaesthesiology and Reanimation, Adana, Türkiye

**Keywords:** Glucagon-like peptide-1 receptor agonists, anaesthesia, gastric emptying, pulmonary aspiration, obesity

## Abstract

Glucagon-like peptide-1 receptor agonists (GLP-1 RAs) and dual glucose-dependent insulinotropic polypeptide GLP-1 RAs are increasingly prescribed for diabetes and obesity, leading to a growing number of surgical patients receiving these agents. Their ability to delay gastric emptying has raised concerns about residual gastric contents (RGCs) and potential aspiration during anaesthesia. Available evidence from mechanistic studies, clinical investigations, and case reports indicates that GLP-1-based therapies consistently impair solid-phase gastric emptying and may increase RGC, particularly during early treatment and dose escalation, with effects that can persist despite standard fasting and short-term drug interruption. Although clinically apparent aspiration events remain uncommon, multiple reports have described perioperative regurgitation or unexpected solid gastric contents at induction. Early guidance favoured routine preoperative drug interruption; however, more recent multisociety recommendations increasingly support continuation of therapy in most asymptomatic patients and endorse enhanced perioperative mitigation strategies, such as dietary modification, strict adherence to fasting, selective use of point-of-care gastric ultrasound, preference for regional anaesthesia when feasible, and tailored airway management. Overall, current data support an individualised, risk-adapted approach rather than uniform interruption of GLP-1 therapy. Continuation of structured mitigation appears reasonable for many patients, whereas heightened caution and full-stomach precautions remain appropriate in higher-risk situations. Further prospective studies are required to define true perioperative aspiration risk and to establish evidence-based management pathways.

**Main Points**

• Glucagon-like peptide-1 (GLP-1) receptor agonists delay solid-phase gastric emptying and may increase residual gastric contents despite standard fasting and short-term drug interruption.

• Clinically evident aspiration appears uncommon; however, perioperative regurgitation and unexpected solid gastric contents have been reported, particularly during early treatment and dose escalation.

• Contemporary multisociety guidance increasingly supports continuation of GLP-1 therapy in most asymptomatic patients, coupled with enhanced perioperative mitigation rather than routine drug interruption.

• Risk-adapted management incorporating dietary modification, strict fasting adherence, selective use of point-of-care gastric ultrasound, and tailored airway and anaesthetic strategies is central to safe perioperative care.

## Introduction

Glucagon-like peptide-1 receptor agonists (GLP-1 RAs) and dual glucose-dependent insulinotropic polypeptide (GIP)/GLP-1 RAs, initially developed for the treatment of type 2 diabetes mellitus, are now widely prescribed for obesity and metabolic diseases owing to their potent effects on glycaemic control, appetite suppression, and weight reduction.^1, 2, 3^ With the rising global prevalence of obesity, perioperative clinicians are increasingly encountering patients receiving agents such as semaglutide, liraglutide, and tirzepatide for both approved and off-label indications.^4, 5^ Although these medications confer substantial metabolic and cardiovascular benefits, they also modify gastrointestinal physiology—most notably by slowing gastric emptying via vagal and peripheral mechanisms—which may result in residual gastric contents (RGC) despite standard fasting, and may raise concern for regurgitation and pulmonary aspiration during anaesthesia.^6, 7, 8^

These concerns are particularly relevant in patients with obesity or type 2 diabetes mellitus, who frequently exhibit baseline vulnerabilities such as gastro-oesophageal reflux, autonomic dysfunction, delayed gastric transit, reduced pulmonary reserve, and increased difficulty in airway management.^9, 10, 11, 12^ The addition of a therapy that further impairs gastric motility may therefore amplify existing perioperative risks and necessitate tailored management strategies. However, the available evidence remains limited.^13^ Current data are derived largely from gastric physiology studies, case reports of peri-induction regurgitation, and small observational cohorts, and there are few controlled clinical trials.^14^ Moreover, findings are heterogeneous: while several studies demonstrate increased retention of solids after standard fasting—sometimes persisting even after treatment interruption—others report minimal or variable effects, depending on dose, duration of therapy, indication, and comorbid conditions.^15^

In keeping with this uncertainty, professional recommendations vary considerably. Early anaesthesia-focused guidance advocated interrupting long-acting GLP-1 RAs prior to elective procedures, based on pharmacokinetic considerations and emerging case reports.^16, 17^ More recent multidisciplinary statements favour an individualised approach incorporating symptom assessment, modified fasting protocols, and selective perioperative gastric evaluation, rather than routine treatment interruption.^18, 19, 20, 21^ Such divergence underscores the absence of consensus and highlights the need to integrate mechanistic, clinical, and guideline-based perspectives.

Accordingly, this narrative review aims to summarise the current evidence on the perioperative implications of GLP-1 RAs, integrate mechanistic insights, clinical studies, and contemporary guideline recommendations, and propose a practical, risk-adapted framework for perioperative management.

### Literature Identification Strategy

A comprehensive literature search was conducted using PubMed, Embase, Web of Science, and Scopus to identify publications relevant to the perioperative management of patients receiving GLP-1 RAs. The search included studies published in English up to February 2026 and encompassed original research articles, clinical studies, case reports, guideline statements, review articles, and perioperative consensus documents. Keywords and search term combinations included “GLP-1 RA,” “GIP/GLP-1 RA,” “semaglutide,” “liraglutide,” “tirzepatide,” “gastric emptying,” “gastric motility,” “gastroparesis,” “RGC,” “perioperative,” “aspiration risk,” “regurgitation,” “airway management,” and “preoperative fasting.” In addition, the reference lists of relevant articles were screened to identify further relevant studies.

### Pharmacology and Mechanisms of Action of GLP-1 RAs

GLP-1 RAs are incretin-based therapies that activate GLP-1 receptors expressed in pancreatic β-cells, the gastrointestinal tract, vagal afferent pathways, and central satiety centres.^22^ In contrast to native GLP-1, which is rapidly degraded by dipeptidyl peptidase-4, pharmacological analogues are engineered with structural modifications—such as albumin binding, peptide acylation, or increased molecular size—to prolong circulation time and resist enzymatic degradation.^23^ These properties permit once-daily or once-weekly administration and result in sustained metabolic effects that are relevant to perioperative care.

### Agent-Specific Pharmacological Profiles

**Semaglutide** (Ozempic®, Wegovy®-subcutaneous weekly; Rybelsus®-oral daily) has an elimination half-life of approximately 6-7 days and reaches steady state after 4-5 weeks of therapy. Complete elimination may require more than five half-lives (approximately 30-35 days). Consequently, clinically meaningful effects on gastric emptying may persist for 2-4 weeks after discontinuation, particularly when discontinued early in therapy.

**Liraglutide** (Saxenda®, Victoza®-subcutaneous daily) has a shorter half-life of approximately 11-13 hours, with near-complete clearance within 3-4 days. Gastric emptying delay is typically mild to moderate during early treatment and tends to diminish with long-term use. Residual effects beyond 48-72 hours are unlikely, although symptomatic patients may remain at increased risk.

**Dulaglutide** (Trulicity®-subcutaneous, weekly) has a half-life of approximately 4.5-5 days, and full elimination may take up to four weeks depending on the dose. Effects on gastric motility are generally modest, becoming more apparent at higher doses or during dose escalation. Delayed gastric emptying may therefore persist for 1-3 weeks after cessation in selected patients.

**Tirzepatide** (Mounjaro®, Zepbound®-subcutaneous weekly; dual GLP-1/GIP RA) has an approximate half-life of five days, reaches steady state after approximately four weeks, and may require four weeks or longer for elimination. Gastric motility effects are less well characterised; however, early data suggest a dose-dependent delay comparable to that observed with long-acting GLP-1 RAs. Pending further evidence, persistence of delayed gastric emptying for 2-4 weeks after discontinuation should be assumed.

**Exenatide** (Byetta®-subcutaneous twice daily; Bydureon®-subcutaneous weekly) is available in short-acting and extended-release formulations: the short-acting formulation has a half-life of approximately 2.4 hours and produces a pronounced postprandial delay in gastric emptying, whereas the extended-release formulation has a half-life of approximately 6-7 days and a less pronounced effect on gastric emptying. Clearance of the twice-daily formulation typically occurs within 24 hours, whereas the extended-release formulation may require three to four weeks for elimination.

### Perioperative Physiologic and Anaesthetic Implications

### Gastric Emptying and Aspiration Risk

Delayed gastric emptying represents the central perioperative concern associated with GLP-1 RAs.^24^ These agents slow gastric motility by reducing antral contractions, increasing pyloric tone, and modulating vagal pathways, which collectively prolong solid-phase retention even when patients adhere to standard fasting instructions.^25^ Consequently, gastric contents may not reliably clear despite 8-12 hours of fasting, particularly in individuals receiving long-acting weekly formulations.^26^

Evidence from case reports, prospective gastric ultrasonography studies, and endoscopic cohorts consistently demonstrates a higher prevalence of retained solid gastric contents among GLP-1 RA users.^27, 28, 29^ Unexpected solid residues have been observed during induction, sometimes accompanied by regurgitation despite appropriate fasting. Although increased RGC raises concern regarding perioperative aspiration risk, this physiological finding does not necessarily translate into a proven increase in clinically evident aspiration. Moreover, the current evidence regarding aspiration risk remains limited and is largely derived from observational studies and case reports.^30, 31^

### Haemodynamic and Endocrine Considerations

Beyond their effects on gastrointestinal motility, GLP-1 RAs also produce endocrine and modest cardiovascular changes that may be relevant in the perioperative setting. These agents lower blood glucose by stimulating insulin secretion in a glucose-dependent manner; therefore, the risk of significant hypoglycaemia is generally low unless they are coadministered with insulin or sulfonylureas.^32, 33^ This profile may facilitate perioperative glycaemic management compared with some other antidiabetic therapies, particularly during prolonged fasting or when caloric intake is uncertain.

**Figure 1:**
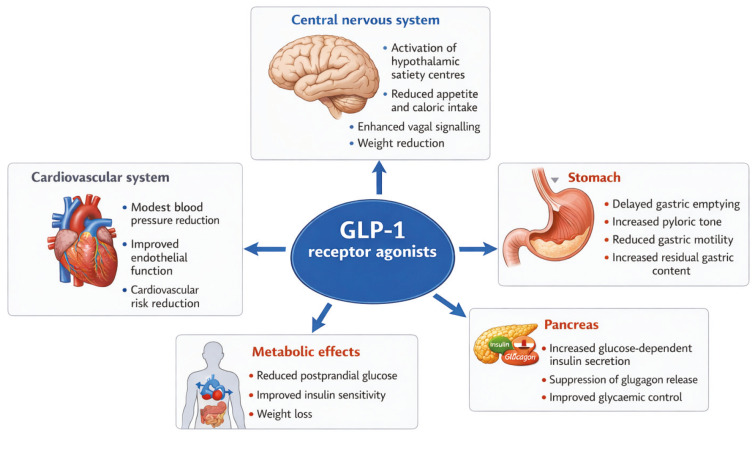
Physiological effects and mechanisms of GLP-1 receptor agonists. GLP-1, glucagon-like peptide-1; GIP, glucose-dependent insulinotropic polypeptide.

Long-term GLP-1 RA therapy may also modestly reduce blood pressure and sympathetic tone through weight loss, natriuresis, and improvement in metabolic parameters.^34^ Clinically significant intraoperative haemodynamic instability directly attributable to GLP-1 RA therapy has not been clearly demonstrated in the perioperative literature; however, the available evidence remains limited. Accordingly, GLP-1 RA therapy alone does not justify routine modification of anaesthetic drug selection or monitoring; perioperative management should be guided by the overall clinical context. These physiological effects and their potential perioperative implications are schematically summarised in Figure 1.

### Evidence from Clinical Studies

### Observational and Interventional Studies

Available clinical evidence regarding the perioperative effects of GLP-1 RAs remains limited, but generally points in a consistent direction. Across prospective gastric ultrasonography studies, retrospective endoscopic series, and mechanistic investigations, GLP-1 RA use is associated with delayed solid-phase gastric emptying and increased RGC, particularly during early treatment phases and periods of dose escalation. Notably, these effects have been observed despite standard fasting protocols and short-term drug interruption, suggesting that routine preoperative fasting may be insufficient in a subset of patients.^26-28,35^ A recent prospective multicentre matched-control study by Vlaeminck et al.^36^ further supports these findings, demonstrating a significantly higher prevalence of a “full stomach” on preoperative gastric ultrasound in semaglutide users compared with matched controls despite guideline-compliant fasting.

Although increased RGC and impaired gastric clearance are frequently documented, clinically apparent aspiration events remain rare. Gastrointestinal symptoms have been reported to be strongly associated with increased RGC, independent of the duration of preoperative drug interruption; emerging data indicate variability among agents, with long-acting formulations and dual agonists conferring higher risk.^28, 37, 38, 39^ An integrated overview of the included clinical studies is presented in Table 1.

### Case Reports and Case Series

Published case reports and small case series provide important early clinical signals regarding the perioperative implications of GLP-1 RAs. Across the literature, delayed gastric emptying, RGC, and perioperative regurgitation or aspiration have been reported despite adherence to standard—and in some cases prolonged—fasting protocols. Although these reports represent low-level evidence, they illustrate scenarios in which routine perioperative precautions failed to ensure gastric emptying, particularly in patients receiving long-acting GLP-1 RAs such as semaglutide.^30, 31, 44, 45^

One illustrative case involved a non-obese, non-diabetic patient who was using semaglutide for weight loss and experienced large-volume regurgitation during induction of anaesthesia after approximately 20 hours of fasting from solid food. Notably, the last semaglutide dose had been administered two days prior to surgery, underscoring the persistence of pharmacological effects beyond both prolonged fasting and short-term drug interruption.^30^ Similar cases have described aspiration events despite residue-free diets and withholding periods of up to six days, suggesting that neither dietary modification nor brief cessation reliably mitigates risk in susceptible individuals.^31, 44^ In other reports, retained solid gastric contents were incidentally identified intraoperatively or on imaging, with previously undisclosed GLP-1 RA use later recognised as the likely contributor.^45^

Additional case series describe reversible drug-induced gastroparesis and severe gastrointestinal dysmotility associated with GLP-1 RA therapy, often following rapid dose escalation or occurring in patients with pre-existing motility disorders. Symptoms—including nausea, vomiting, abdominal pain, and delayed transit—consistently improved after drug discontinuation, supporting a causal and potentially reversible effect.^46, 47, 48^ Rare pulmonary complications, such as silent microaspiration and organising pneumonia, have also been reported, indicating that delayed gastric emptying may have clinically relevant consequences even outside the immediate perioperative setting.^49^

### Guideline and Consensus Recommendations

Early guidance on the perioperative management of GLP-1 RAs was driven largely by precaution rather than high-quality evidence. The American Society of Anesthesiologists (ASA) 2023 consensus-based guidance was the first formal response to emerging case reports of regurgitation and aspiration associated with long-acting GLP-1 agents. Acknowledging the very low quality of available evidence, ASA adopted a conservative, safety-first strategy centred on preoperative drug withholding, recommending interruption of daily agents on the day of surgery and of weekly agents for seven days before elective procedures.^17^ Symptomatic patients were advised to be managed as though having a full stomach; gastric ultrasound or full-stomach precautions were suggested if withholding was incomplete.

Subsequent European guidance introduced greater nuance. The European Society of Anaesthesiology and Intensive Care (ESAIC) 2025 guideline, developed using a structured patient/population, intervention, comparison, outcome framework, retained drug withholding as a core strategy but emphasised patient- and procedure-specific risk stratification.^18^ While recommending the interruption of weekly agents for at least one week—and up to two weeks in high-risk contexts such as obesity or bariatric surgery—the guideline explicitly recognised that even prolonged interruption may not normalise gastric emptying. Compared with ASA, ESAIC placed greater emphasis on mitigation strategies, including point-of-care gastric ultrasound, clear-liquid diets for selected patients, and airway-protection measures when aspiration risk was suspected.

More recent multidisciplinary statements have shifted away from routine drug interruption towards continuation-focused strategies. The Society for Perioperative Assessment and Quality Improvement 2025 consensus, the Australian-New Zealand joint recommendations led by the Australian and New Zealand College of Anaesthetists, and the 2025 United Kingdom multisociety consensus endorsed by the Association of Anaesthetists, all concluded that the evidence linking GLP-1 continuation to clinically significant aspiration is weak, whereas the metabolic and cardiovascular consequences of drug interruption are well established.^19, 20, 21^ These documents favour the continuation of GLP-1 and dual GIP/GLP-1 RAs in most patients, with risk mitigation achieved through dietary modification, strict adherence to fasting, selective use of gastric ultrasound, and tailored anaesthetic and airway strategies, rather than by routine interruption. The key recommendations, conceptual differences, and risk-mitigation strategies across major guidelines and consensus statements are summarised in Table 2.

### Current Clinical Recommendations and Practical Considerations

Clinicians are increasingly encountering patients treated with GLP-1 RAs and dual GIP/GLP-1 RAs, while the perioperative evidence base remains indirect and heterogeneous. Consequently, current recommendations do not reflect a single universal standard but rather an evolving synthesis across professional societies, largely informed by observational data and expert consensus.

There is broad agreement that GLP-1 RAs delay solid-phase gastric emptying and increase RGC; however, the clinical relevance of these physiological changes in terms of aspiration risk remains uncertain. This uncertainty underlies the observed divergence in guidance. The ASA and ESAIC adopt a precautionary approach, recommending temporary interruption of daily agents on the day of surgery and of weekly formulations for at least one week, while acknowledging that cessation may neither normalise gastric emptying nor be metabolically benign (a consensus-based recommendation).

In contrast, more recent multisociety statements favour continuation of GLP-1 therapy in most asymptomatic patients, emphasising that prolonged interruption rarely restores gastric motility and may expose patients to hyperglycaemia, weight rebound, and loss of cardio-renal benefit. These groups instead prioritise mitigation strategies, including strict adherence to fasting, selective use of a 24-hour clear-fluid diet, regional anaesthesia, when feasible, and point-of-care gastric ultrasound for individualised risk assessment. However, gastric point-of-care ultrasound is operator-dependent and its availability may be limited in some perioperative settings. These recommendations are supported mainly by observational data and expert opinion.

Across all guidance, symptomatic patients and those in early dose-escalation phases are consistently identified as higher-risk groups, requiring heightened caution, postponement of procedures, or precautions related to a full stomach. When uncertainty persists, full-stomach precautions with appropriate airway protection remain the default. Overall, contemporary practice is shifting from routine drug interruption toward a continuation-plus-mitigation model that balances the uncertain aspiration risk against the clearer metabolic harms.

### Proposed Perioperative Management Strategy

This proposed approach is informed by clinical experience in the perioperative care of patients with obesity, in whom airway management is often more complex and tolerance for aspiration-related complications is limited.

From an anaesthesiology perspective, the possibility of a “full stomach” remains a central concern, particularly when associated with factors such as obesity. Although documented aspiration events are uncommon, reports of regurgitation and aspiration with solid gastric contents—even after prolonged fasting—suggest that this risk cannot be considered purely theoretical.^30, 31^

In the context of an evolving, evidence-limited literature, patient safety should remain the primary consideration. Increased perioperative awareness of GLP-1 and dual GIP/GLP-1 RA therapy among patients and healthcare providers is essential, as incomplete disclosure or under-recognition of these agents may contribute to avoidable risks.

Decisions regarding continuation or temporary interruption of therapy should be individualised and, when feasible, involve multidisciplinary input from anaesthesiology, endocrinology, and cardiology. For elective procedures, a longer interruption period and enhanced dietary preparation may be reasonable in selected high-risk patients, while recognising that drug interruption alone may not reliably normalise gastric emptying. Accordingly, such patients should continue to be approached with a degree of caution regarding aspiration risk.

**Figure 2:**
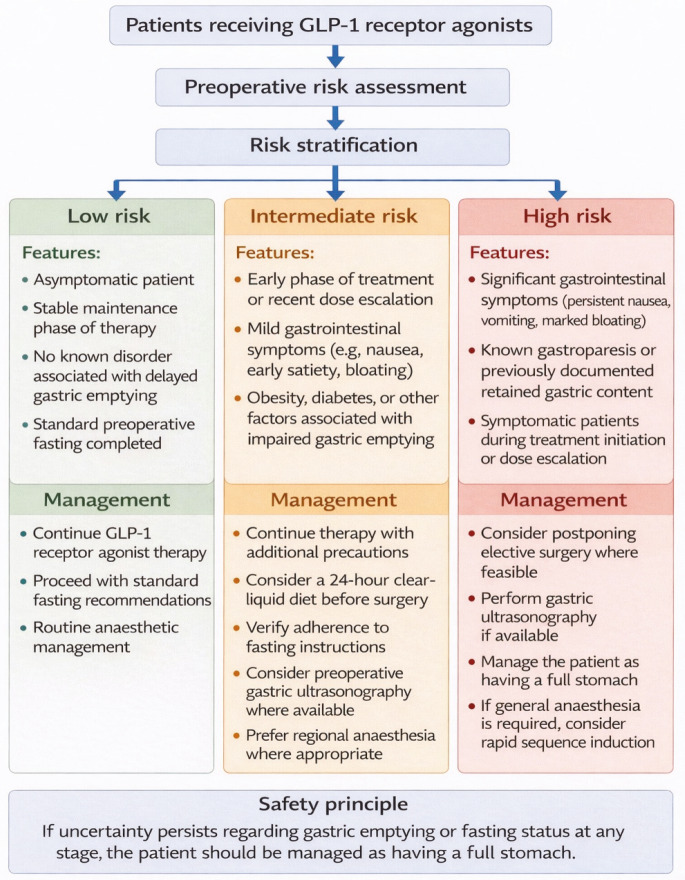
Proposed risk-adapted perioperative management algorithm for patients receiving GLP-1 and dual GIP/GLP-1 receptor agonists. GLP-1, glucagon-like peptide-1.

When discontinuation is not feasible—such as in urgent or emergent settings—risk mitigation becomes central. Regional anaesthesia should be preferred when appropriate. If general anaesthesia is required, strategies may include rapid sequence induction, tracheal intubation, selective use of prokinetics or gastric decompression, minimising opioid exposure, and close postoperative monitoring. Overall, this approach aligns with an emerging consensus favouring individualised mitigation-based perioperative management rather than uniform drug interruption, thereby balancing aspiration risk against the metabolic benefits of continued therapy. A proposed risk-adapted perioperative management algorithm is presented in Figure 2.

### Clinical and Research Consequences

The expanding use of GLP-1 RAs and dual GIP/GLP-1 RAs has important implications for everyday anaesthetic practice. Current evidence supports a shift away from uniform drug interruption towards an individualised, risk-adapted approach that integrates patient symptoms, treatment phase, comorbidities, and procedural risk. For clinicians, this necessitates heightened awareness of these agents, routine preoperative enquiry about their use, and consideration of mitigation strategies, such as dietary modification, regional anaesthesia, when feasible, and selective application of point-of-care gastric ultrasound.

From a research perspective, substantial knowledge gaps remain. Prospective studies evaluating the true incidence of perioperative aspiration, the duration of gastric emptying impairment after drug interruption, and the comparative risks associated with different GLP-1 and dual agonist formulations are needed. Further studies should explore whether perioperative continuation or interruption of therapy influences glycaemic stability, cardiovascular outcomes, and postoperative complications. Addressing these gaps will be essential for developing evidence-based, standardised perioperative management pathways.

### Study Limitations

This review has several limitations. First, it was conducted as a narrative rather than a systematic review, reflecting the limited and evolving nature of the available evidence in this field. Second, much of the current knowledge regarding the perioperative implications of GLP-1 RAs is derived from physiological studies, case reports, and relatively small observational cohorts rather than large prospective clinical trials. Finally, differences in study populations, drug formulations, treatment duration, and perioperative protocols contribute to substantial heterogeneity across the available literature.

## Conclusion

Current evidence regarding the anaesthetic implications of GLP-1 RAs remains limited and largely observational. Available data suggest that delayed gastric emptying and increased RGCs may persist despite standard fasting and short-term drug interruption; however, the clinical significance of these findings in terms of aspiration risk has not yet been clearly defined. Recent multisociety guidance increasingly supports continuation of therapy in selected patients, although this approach is consistently coupled with enhanced perioperative risk-mitigation strategies rather than unconditional continuation of therapy.

Accordingly, perioperative management should be individualised and guided by multidisciplinary input, particularly in patients with obesity, diabetes, or additional risk factors for impaired gastric emptying. In lower-risk, asymptomatic patients, continuation of GLP-1 therapy may be reasonable provided that extended solid-food restriction, clear-liquid dietary preparation, and strict adherence to fasting are observed. In higher-risk situations or when uncertainty persists, heightened caution—including full-stomach precautions and selective use of point-of-care gastric ultrasound—remains appropriate. Until prospective data more precisely define true perioperative risk, a risk-adapted strategy combining therapy continuation with structured mitigation represents a balanced and defensible approach.

## Figures and Tables

**Table 1. Summary of Clinical Studies on Perioperative Effects of GLP-1 RAs:** 

**Study/year**	**Population**	**Method of assessment**	**Key findings**	**Clinical implication**
**Sherwin et al.^26^**	Volunteers without obesity recently started on semaglutide	Gastric ultrasound after ≥10 h overnight fasting	Retained solids in 70-90% of semaglutide users vs. 10-20% of controls	Early semaglutide use causes marked solid-phase delay even in non-obese subjects
**Nersessian et al.^27^**	Elective surgery patients who used SG≤10 days preop. No diabetes, no obesity	Preoperative point-of-care gastric ultrasound	Increased RGC in 40% vs. 3% of controls; younger male patients at highest risk	Semaglutide within 10 days markedly increases RGC; even 10-day cessation may be insufficient
**Jensterle et al.^35^**	Obese women with PCOS	Gastric scintigraphy with solid meal	37% retention at 4 h vs. 0% in controls; T½ prolonged from 118 ® 171 min	Mechanistic evidence in obese endocrine population
**Vlaeminck et al.^36^**	Elective surgery patients receiving semaglutide vs. matched controls	Prospective multicentre gastric ultrasound study	Full stomach: 49% vs. 18% (OR 4.29); solids 42% vs. 7%	Semaglutide associated with increased RGC despite guideline-compliant fasting
**Gabe et al.^37^**	Adults with obesity	Paracetamol absorption (liquid emptying surrogate)	No change in gastric emptying; no delay detected	Possible tachyphylaxis; liquid-phase tests may underestimate effects
**Silveira et al.^38^**	EGD under sedation/general anaesthesia SG use within 30 days	Direct endoscopic measurement of RGC (solids or >0.8 mL kg^-1^ fluid)	Increased RGC 24.2% vs. 5.1%; interruption interval irrelevant	GLP-1 RA users show higher RGC despite ~10-day drug withdrawal
**Santos et al.^28^**	Adults undergoing EGD under deep sedation/general anaesthesia	Endoscopic quantification of residual gastric content (solids or >0.8 mL kg^-1^ fluid)	Higher RGC (20.3% vs. 3.2%); GI symptoms key predictor; risk persists if semaglutide withheld ≤14 days, normalizing only after >14-21 days.	Perioperative semaglutide markedly increases RGC withholding >14-21 days may be required
**Robalino Gonzaga et al.^39^**	Ambulatory EGD cohort	Direct endoscopic visualization of solid food retention	GLP-1 use ­ RGC risk ~9-fold; tirzepatide highest (45%)	Strong association between GLP-1 use and RGC; but aspiration remains rare
**Dahl et al.^40^**	Well-controlled T2D adults	Paracetamol absorption test (liquid-phase GE surrogate)	Early gastric emptying reduced by 31%, with no delay observed in the later 5-hour phase	Only early liquid-phase GE is delayed; no sustained GE delay demonstrated
**Muranaka et al.^41^**	Elective surgery patients taking semaglutide <7 days pre-op	Gastric POCUS; solid content or >1.5 mL kg^-1^ =“full stomach”	20% had RGC. 1-3 day interval ® 75% full stomach, 4-6 day interval ® 10%. Dose/BMI/diabetes not predictive	G-POCUS prevents unnecessary cancellations; semaglutide within <3 days carries highest RGC risk
**Skidmore et al.^42^**	Diabetics with GLP-1 using ambulatory urology surgeries	Postop DSpO_2_ as surrogate for micro-aspiration/atelectasis	GLP-1 hold <7 vs. ≥7 days ® no difference in DSpO_2_	GLP-1 hold duration does not impact pulmonary risk
**Sen et al.^43^**	Fasted elective surgery patients; weekly GLP-1 RA users vs. controls	Gastric ultrasonography; solids, thick liquids or >1.5 mL kg^-1^=increased RGC	RGC: 56% vs. 19%; GLP-1 use ­ risk (adjusted prevalence ratio 2.48); no association between stop duration (≤7 days) and RGC	GLP-1 RA is independent risk factor for high RGC. Stopping for 7 days does not normalize, GUS-based assessment recommended

RGC, residual gastric content; EGD, esophagogastroduodenoscopy; GI, gastrointestinal; PCOS, polycystic ovary syndrome; T2D, type 2 diabetes; GE, gastric emptying; POCUS, point-of-care ultrasound; GUS, gastric ultrasonography; RLD, right lateral decubitus; DSpO_2_, difference between preoperative and postoperative oxygen saturation; SG, semaglutide group; NSG, non-semaglutide group; RA, receptor agonist; GLP-1, glucagon-like peptide-1; OR, odds ratio

**Table 2. Comparative Summary of Major Guidelines and Multisociety Consensus Statements on Perioperative Management of GLP-1 and GIP/GLP-1 RAs:** 

**Feature**	**ASA 2023**	**ESAIC 2025**	**SPAQI 2025**	**ADS/ANZCA/GESA/NACOS 2025**	**United Kingdom multisociety 2025**
Document type	Consensus-based guidance (expert opinion)	Formal guideline with clinical practice suggestions (CPS)	Multidisciplinary consensus with graded recommendations	Multisociety practice recommendations (conditional GRADE)	Multidisciplinary consensus (Delphi-based)
Evidence appraisal	Narrative evidence from case reports and small observational series; no GRADE	Structured PICO; CPS issued due to low-quality evidence	Systematic review + modified Delphi; graded (B, C, E)	Abbreviated Delphi; conditional GRADE	Directed review and three-round Delphi; largely observational data
Overall evidence quality	Very low	Low	Low to moderate	Low	Low
Perioperative GLP-1 strategy	Withhold: daily agents on DOS; weekly agents 7 days prior	Withhold: daily on DOS; weekly ≥1 week (up to 2 weeks for high-risk patients)	Continue in asymptomatic patients	Continue routinely; cessation generally not advised	Continue perioperatively; avoid routine cessation
Fasting/diet strategy	Standard ASA fasting; no changes recommended	Standard fasting; consider 24-h clear-liquid diet in high-risk patients	Extend solid-food restriction to 24 h; carbohydrate-adjusted clear liquids	Universal 24-h clear-liquid diet and standard fasting	Standard national fasting; emphasise strict adherence
Core risk concept	Aspiration risk mitigated primarily through drug interruption and full-stomach precautions	All GLP-1 users at potential risk; amplified by obesity, diabetes, bariatric surgery	Aspiration risk small and uncertain; continuation favored due to metabolic benefit	Delayed emptying persists despite brief cessation; focus on diet with procedural mitigation	Aspiration risk real but small; metabolic destabilization outweighs theoretical benefit of withholding
Risk stratification	Symptom-based (nausea, vomiting, abdominal discomfort)	Indication- and risk-profile based (obesity, diabetes, bariatric surgery, agent type)	Based on symptom burden, comorbidities, and procedure type	Fasting completeness, procedure type, adherence to clear-fluid protocol	Integrated: drug (dose/timing), patient (diabetes/obesity/gastroparesis), procedure/anaesthetic factors
Role of gastric ultrasound	Optional if drug not withheld; informs full-stomach precautions	Strongly encouraged, if RGC present ® consider postponement	Mentioned but not central; more emphasis on fasting modification	Key tool if clear-fluid protocol not followed; alternative is ultrathin endoscopy	Recommended where appropriate; guides airway planning and aspiration risk
Airway/anaesthetic mitigation	Full-stomach precautions; consider RSI and tracheal intubation	RSI/intubation in high-risk patients; full-stomach approach if ultrasound unavailable	Mitigation tailored to overall risk; no universal RSI	Prefer regional anaesthesia with minimal sedation; RSI/full-stomach precautions when risk high	Prefer regional anaesthesia; selective use of RSI, prokinetics, gastric decompression, head-up position
Cardiometabolic impact of cessation	Acknowledged mainly for diabetic patients; endocrinology input advised	Briefly discussed; less emphasis than others	Strongly emphasized (hyperglycemia; loss of cardio-renal protection)	Highlighted (hyperglycemia, weight and BP destabilization)	Strong emphasis on hyperglycemia, stress hyperglycemia, loss of long-term cardio-renal benefit
Conceptual stance	Precautionary, drug-withholding, aspiration-focused	Risk-tailored withholding with procedural mitigation	Continuation-forward with fasting modification	Continuation and strengthened mitigation (diet/imaging/procedure-level)	Continuation-focused, system-level risk stratification and shared decision-making

ASA, American Society of Anesthesiologists; ESAIC, European Society of Anaesthesiology and Intensive Care; SPAQI, Society for Perioperative Assessment and Quality Improvement; ADS, Australian Diabetes Society; ANZCA, Australian and New Zealand College of Anaesthetists; GESA, Gastroenterological Society of Australia; NACOS, National Association of Clinical Obesity Services; GLP-1, glucagon-like peptide-1; GIP, glucose-dependent insulinotropic polypeptide; RGC, residual gastric content; RSI, rapid sequence induction; CPS, Clinical Practice Suggestions; DOS, day of surgery; PICO, patient/population, intervention, comparison, outcome
